# Long term outcome of otosclerosis surgery

**DOI:** 10.1590/S1808-86942012000400021

**Published:** 2015-10-20

**Authors:** Maria Teresa Bernardo, Joana Dias, Daniela Ribeiro, Diamantino Helena, Artur Condé

**Affiliations:** 1 MD (Medicine/ENT) (Intern ENT - CHVNG/E.EPE); 2 MD (Audiology) (Audiology technician - CHVNG/E.EPE); 3 MD (Medicine/ENT) (Senior ENT Assistant ORL - CHVNG/E. EPE); 4 MD (Medicine/ENT) (Head of the ENT Service - CHVNG/E.EPE)

**Keywords:** otosclerosis, stapes surgery, audiology

## Abstract

The treatment of otosclerosis is eminently surgical. Good immediate results have been well documented when stapedotomy or stapedectomy are chosen.

**Objectives**: This study aims to assess long term audiometric performance after otosclerosis surgery.

**Materials and Methods**: this retrospective study enrolled stapedotomy and partial stapedectomy patients seen at our service with proven hearing improvement after surgery. Forty-one patients (47 ears) accepted the invitation to be reassessed. Audiometry results before and immediately after surgery were compared.

**Results**: the median late follow-up was 11 years. To this date, 49% of the patients had normal hearing or mild dysacusis. Preoperative, postoperative, and late postoperative bone and air pure tone averages were 64.4 and 27.0 dB, 35.6 and 22.3 dB, and 44.1 and 29.5 dB respectively.

**Conclusion**: Otosclerosis surgery offers good long term results. Despite the worsening of thresholds, the level of hypacusis ten years after surgery is lower than the levels observed before surgery.

## INTRODUCTION

Otosclerosis is a disease that involves the otic capsule. It stems from altered bone metabolism and is characterized by the presence of various foci of bone resorption and bone replacement^1^, ^2^, ^3^, in a structure that, under normal circumstances, shows no osteoclastic or osteoblastic activity^1^, ^4^, ^5^. Otosclerosis was first described by Valsava in 1741, from an autopsy done on a deaf subject with stapedial ankylosis. In 1894, Politzer defined otosclerosis as a clinical entity^1^, ^4^. Despite the countless studies on this subject, the etiology of otosclerosis still remains a mystery. Most authors consider it to be a multifactorial disease^4^. There seems to be genetic predisposition with autosomal dominant inheritance, incomplete penetrance (40%), variable expressivity^4^, and even sporadic onset (40%-50%)^6^.

The most commonly mentioned triggering factors are the measles virus, hormonal alterations (e.g.: pregnancy, phosphocalcic metabolism) and autoimmunity^4^. Approximately 10% of the Caucasian population has histological traits of otosclerosis, but only 10% of this group will present clinical signs of the disease^1^, ^4^. Otosclerotic plaques are more frequently found in the region anterior to the oval window (80%-95%)^7^ and when they reach it they condition the fixation of footplate and consequently introduce transmission hearing loss. Surgery is the current treatment of choice. Stapedectomy or stapedotomy^2^, ^8^ are the preferred procedures, the latter being the most frequently used^9^ as it produces fewer complications^10^, ^11^, ^12^, ^13^, namely less worsening of of bone thresholds at high frequencies^1^. Good immediate outcome has been reported for both procedures, but long term results are frequently questioned. Some studies have reported stable bone thresholds after surgery, while others show sensorineural hearing loss of varying degrees along the years^9^. In most cases hearing loss was related to age^14^, ^15^, ^16^, ^17^, but it may originate from cochlear endosteum involvement caused by cochlear otosclerosis^5^, ^9^.

Conventional hearing aids are a safe alternative and offer results comparable to those of surgery in patients with pure transmission hearing loss^1^, ^5^, although the same cannot be said of mixed hearing loss cases^6^. Hearing aids may be needed as salvage treatment some years (13 to 30) after surgery^18^, ^19^. Osseointegrated hearing implants do not imply in footplate fenestration, and thus eliminate the risk of cophosis^1^, ^6^. This study aims to assess audiometric test outcomes 10 years after otosclerosis surgery in patients with recorded immediate postoperative results.

## MATERIALS AND METHODS

This is a retrospective study including stapedotomy and partial stapedectomy patients from our service operated on from November of 1998 to January of 2002. The diagnosis of otosclerosis was confirmed during surgery.

Postoperative hearing improvements were backed up by pure-tone audiometry. Revision surgery cases and patients with complications were excluded. The selected patients were invited for audiometric reassessment and otological examination. They were asked to provide personal data, information on the disease, the procedure and its respective clinical processes.

The values gathered were compared (thresholds at 500, 1000, 2000, 4000, and 8000 Hz) to the preoperative test results. Air bone *gaps* (ABG) and mean ABG were calculated for 500, 1000, 2000 and 4000 Hz, and the pure tone average (PTA) for air and bone thresholds for the same frequencies. The PTA-related Guidelines from the Committee on Hearing and Equilibrium^20^ were not complied with in four frequencies, as the 3000 Hz band was not included in preoperative and immediate postoperative audiograms.

Air and bone thresholds between both ears of patients with bilateral otosclerosis and stapedial surgery were also compared after correction for the Carhart effect (5 dB at 500 Hz, 10 dB at 1000 Hz, 15 dB at 2000 Hz and 5 dB at 4000 Hz)^21^ in the non-operated ears. Contralateral ears were considered to have otosclerosis if otoscopic examination were normal and if there was transmission or mixed hearing loss with at least 10 dB ABG in two or more frequencies or 15dB in one frequency.

The data obtained was statistically treated using Excel 2007 and SPSS 17.0. Student's *t*-test was used to compare paired samples of preoperative, immediate postoperative (postoperative 1) and late postoperative (postoperative 2) audiometry tests; the Wilcoxon test was used when the variable's distribution was not normal. Student's t-test was used to compare the two ears of one same patient for independent samples; otherwise the Mann-Whitney test was used. A statistical significance level of 0.01 was chosen (α= 0.01).

Stapedotomy and partial stapedectomy procedures were carried out with the patient under general anesthesia via the transcanal or endaural approach. A horizontal incision is made on the canal skin 5-6 mm from the annulus and another two vertical incisions are made at 12 and 6 hours; a tympanomeatal flap is produced to allow access to the middle ear. In order to ensure better control over the oval window, the canal's posterosuperior wall is curetted to spare the chorda tympani. After confirming the fixation of the stapes, a safety platinotomy is performed, along with incudostapedial dislocation, sectioning of the stapedius, fracturing of the stapes suprastructure, broadening of the platinotomy/platinectomy, and placement of a Causse-type polytetrafluoroethylene (Teflon®) implant, 0.4-0.6 mm thick and 4.5 to 5.5 mm long. Stapes surgery was indicated for patients with ABG equal to or greater than C30 dB with negative Rinne (512 Hz).

## RESULTS

Fifty-four patients met the enrollment criteria and were invited to do an audiometric reassessment. Forty-one of them (47 ears) showed up, 77% (36/47) being females and all Caucasians. Mean ages at the time of diagnosis, surgery and reassessment were 40.4 ± 8.3 (78.7% in the 30-50 year-old range), 43.8 ± 9.2, and 55.2 ± 9.1 years respectively. The observed symptoms were bilateral hearing loss (61.7%), isolated hearing loss (29.8%), and hearing loss accompanied by tinnitus (31.9%). Median symptom duration was 72 months (1-300 months). Stapedotomy was the most frequently offered surgery (72.3%); the surgical team was made up by a specialist head surgeon in 65.9% of the cases; a 4.5 mm long implant was used in 69.2% of the cases. Forty-two percent (17/41 patients; 23/47 ears) of the patients had contralateral ear surgery. From the 24 non-operated contralateral ears, 10 had otosclerosis, thus yielding 65.9% (27/41 patients; 33/47 ears) of patients with bilateral otosclerosis. Family history of hearing loss (presumably otosclerosis) was observed in 42.6% of the patients.

### Air thresholds

Postoperative and late postoperative median audiometric follow-up were performed at 4 months (1-19 months) and 11 years (10-14 years) respectively. Otoscopic examination was normal for all reassessed ears after ear wax removal. None of the patients had fitted hearing aids in their operated ears. Only 14.6% (6/41) of the patients reported subjective worsening of hearing on late follow-up.

**Figure 1:**
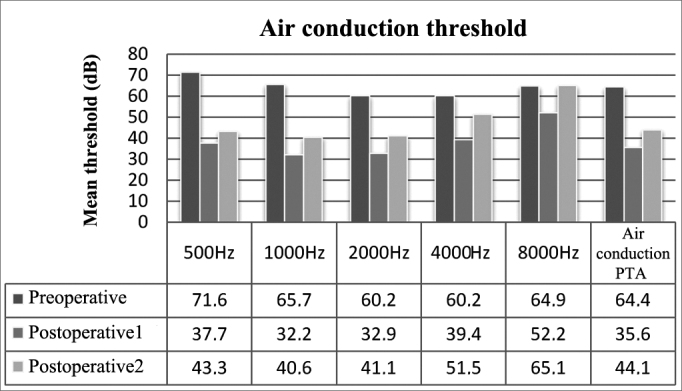
Mean air thresholds for each frequency and air PTA values.

In preoperative air thresholds, the most involved frequencies were 500 and 1000 Hz, a typical sign of otosclerosis. Thresholds were improved in all frequencies immediately after surgery (*p* < 0.01). The comparison between immediate postoperative and late postoperative thresholds revealed hearing worsening (*p* < 0.01) yet not reaching preoperative levels (*p* < 0.01), except at 8000 Hz (*p* > 0.01) (Figure 1).

Preoperative, postoperative, and late postoperative air PTA values were 64.4 ± 15.1 dB, 35.6 ± 11.8 dB, and 44.1 ± 14.1 dB respectively. These values represent a mean gain of 28.8 dB from preoperative to immediate postoperative conditions (*p* < 0.01) and an overall postoperative decrease of 8.5 dB (*p* < 0.01). Late postoperative air PTA levels were lower than preoperative values (*p* < 0.01).

### Bone thresholds

Preoperative bone thresholds indicated greater involvement at 2000 and 4000 Hz (Carhart's scotoma). Thresholds were improved (corrected for Carhart effect) when preoperative and immediate postoperative conditions were compared; statistically significant difference was not observed only at 4000 Hz. Worsening of all bone thresholds were seen in late postoperative reassessment (*p* < 0.01), and values were worse than in preoperative conditions for all frequencies (*p* > 0.01).

**Figure 2:**
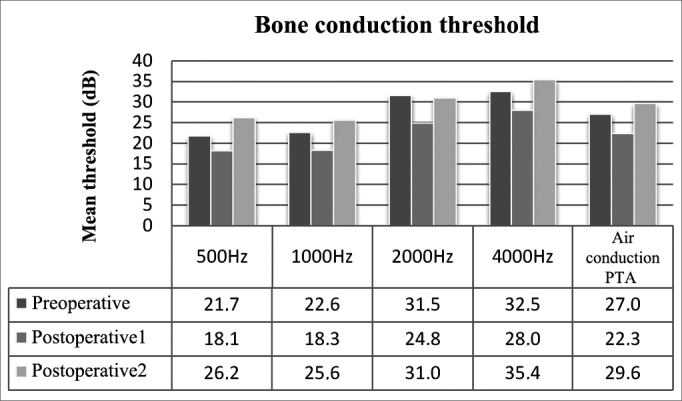
Mean bone thresholds for each frequency and bone PTA values.

Preoperative, postoperative, and late postoperative bone PTA values were 27.0 ± 15.2 dB, 22.3 ± 11.3 dB, and 29.6 ± 12.5 dB respectively, showing a gain of 4.7 dB (*p* < 0.01) from preoperative to immediate postoperative conditions, followed by a global loss of 7.3 dB (*p* < 0.01). Late postoperative bone PTA levels were greater than preoperative values (*p* > 0.01) (Figure 2).

### Air-bone *gap* (ABG)

**Figure 3:**
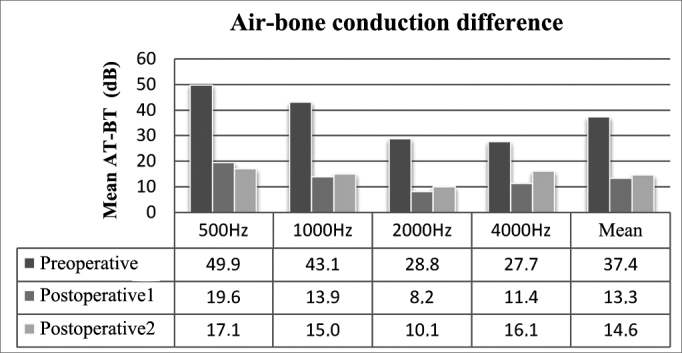
Air-bone *gap* for each frequency and mean ABG values. AT - air threshold; BT - bone threshold.

ABG improved in all frequencies on immediate postoperative care (*p* < 0.01). ABG changed with time, and tended to increase at 1000, 2000, and 4000 Hz, albeit not statistically significant except for 4000 Hz. Mean preoperative, postoperative, and late postoperative ABG values were 37.4 ± 9.3, 13.3 ± 5.7, and 14.6 ± 5.6 dB respectively, showing a mean postoperative gain of 24.1 dB (*p* < 0.01) and a total increase during late follow-up of 1.3 dB (*p* > 0.01). Mean late postoperative ABG did not meet preoperative levels (*p* < 0.01) (Figure 3).

### Mean annual decrease

Air thresholds (AT) had a mean annual decrease (late postoperative AT - immediate postoperative AT/years of follow-up) after surgery of 0.47, 0.74, 0.70, 1.06, and 1.10 dB at 500, 1000, 2000, 4000, and 8000 Hz.

Bone thresholds (BT) had a mean annual decrease (late postoperative BT - immediate postoperative BT/years of follow-up) after surgery of 0.4, 0.64, 0.53, and 0.65 at 500, 1000, 2000, and 4000 Hz.

**Figure 4:**
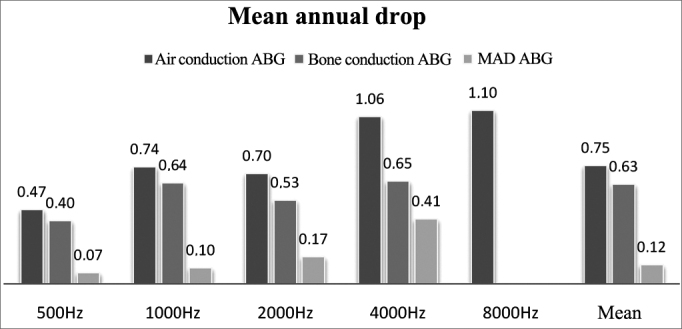
Mean annual decrease (MAD) of air and bone thresholds, and air-bone *gap* (ABG) for each frequency. Mean values correspond to the decrease in air and bone PTA and mean ABG values.

Air and bone PTA annual decreases were 0.75 and 0.63 dB respectively. ABG mean annual increase (mean immediate postoperative ABG - late postoperative ABG/years of follow-up) was 0.12 dB (Figure 4).

### Degree of hypacusis

**Figure 5:**
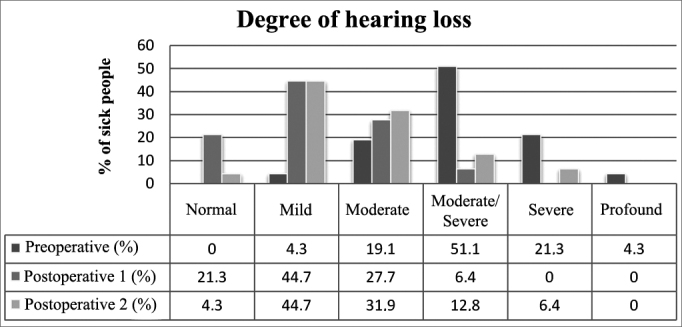
Degree of preoperative, postoperative (1) and late postoperative (2) hypacusis.

Hypacusis was improved immediately after surgery (*p* < 0.01) and got worse along the years without reaching preoperative levels (*p* < 0.01). On late follow-up, 49% of the patients had normal hearing or mild hypacusis (normal (0-25 dB) 4.3% and mild hearing loss (26-40 dB) 44.7%) (Figure 5).

### Comparing ears of patients with bilateral otosclerosis and patients with only one operated ear (n = 20)

**Figure 6:**
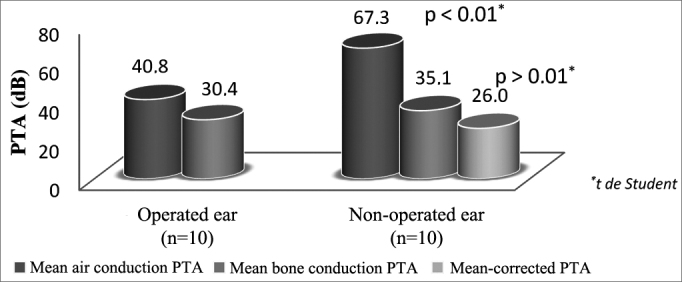
Comparison of air and bone PTA values of patients with bilateral otosclerosis and unilateral stapedial surgery.

Air PTA values of operated and non-operated ears with otosclerosis on late follow-up were 40.8 ± 14.1 and 67.3 ± 16.0 dB respectively with statistically significant difference. Bone PTA values were 30.4 ± 11.5 and 35.1 ± 12.4 dB (26.0 ± 12.7 dB after Carhart correction) in operated and non-operated ears without statistically significant difference (Figure 6).

### Comparing groups

No differences were seen when the postoperative results of both genders, the presence of family history, exposure to noise, type of surgery, makeup of the surgical team (specialist/non-specialist), and hearing aid lengths were compared. ABG closure is greater (*p* > 0.01) in patients over 40, but air PTA is worse (*p* > 0.01).

## DISCUSSION

The higher incidence rates seen in female patients, the presented symptoms, age at the time of diagnosis and surgery, bilateral involvement in 65.9% of the cases, and presence of family history in 42.6% of the patients are findings consistent with the literature^1^, ^2^, ^4^, ^6^, ^8^, ^9^.

The comparison between preoperative and immediate postoperative values escapes the purpose of this paper, as the selected patients had to have documented audiometric improvement, a requirement that introduces sampling bias.

Air thresholds degrade along the years in all frequencies, and even more so at higher frequencies (4000 and 8000 Hz), as described in the literature^2^, ^8^. This occurrence may be translated into acoustic trauma during surgery due to endolymph aspiration^8^ or as the outcome of the aging of the cochlea. These values do not reach preoperative levels. Bone thresholds and ABG also worsen with the years. Bone thresholds can reach and even surpass preoperative values although not in a statistically significant manner, unlike ABG, as it keeps at much lower levels than preoperative values.

The annual decrease in air PTA described in the literature ranges between 0.6 and 2.1 dB^6^, ^22^. Our study found a decrease of 0.75 dB/year greatly due to bone decrease (0.63 dB/year) and not ABG (0.12 dB/year), consistent with what can be expected for aging (loss of 1 dB/year^23^) according to some authors^4^, ^15^, ^16^, ^17^, albeit a controversial topic. In order to make such a statement, we would have to compare this group to a control group without ear disease with the same distribution in terms of age and gender.

On late follow-up, 49% of the patients had normal hearing or mild hypacusis and therefore did not require hearing aids. Only 14.6% of the patients reported subjective hearing worsening, but none wore hearing aids in their operated ears.

When comparing the ears of ten patients with bilateral otosclerosis submitted to surgery on only one ear, we found that 11 years later the air PTA on the operated ears (the one that would initially be considered the worse) was significantly better than that of the contralateral ear. Bone PTA worsens when the correction for Carhart is applied, but not in a statistically significant manner, unlike what was found in other papers in which surgery was found to lead to faster progression of sensorineural hearing loss^9^.

Stapedial surgery may be subject to complications, and they were not taken into account in this study. Nonetheless, surgery seems to be a therapeutical option that offers more longstanding results than hearing aids which, in 10 years' time, would have probably been replaced at least once due to disease progression. A randomized trial is required in order to compare the effectiveness of both therapies^6^.

No statistically significant differences were observed in group comparisons, possibly due to the limited size of the sample. The differences seen in other studies in relation to age ranges and worse prognosis for younger individuals^2^ were not observed in this study, probably because of the limited size of our sample.

## CONCLUSION

Successful otosclerosis surgery offers long-term benefits to patients. Despite the decrease in thresholds seen more often in higher frequencies, there was less hearing loss 11 years after surgery than before it. A mean postoperative decrease of 0.75 dB (mainly due to bone degradation) was observed, similarly to what had been calculated for presbycusis. Surgery improved auditory function of operated ears in the long term when compared to non-operated ears, and stands as a mode of treatment to consider against hearing aids. The decision on mode of treatment belongs to the patient after proper clarification has been provided.
